# *Klebsiella pneumoniae *with capsule type K64 is overrepresented among invasive disease in Vietnam

**DOI:** 10.12688/f1000research.52799.2

**Published:** 2025-01-17

**Authors:** Bich Vu Thi Ngoc, Sylvain Brisse, Trinh Dao Tuyet, Dung Vu Tien Viet, Kathryn E Holt, Trung Nguyen Vu, Huong Tran Thi Kieu, Diep Nguyen Thi Ngoc, H Rogier van Doorn, Heiman F L Wertheim

**Affiliations:** 1 Oxford University Clinical Research Unit - Hanoi, Hanoi, Vietnam; 2 French National Centre for Scientific Research, Paris, France; 3 Microbial Evolutionary Genomics, Institut Pasteur, Microbial Evolutionary Genomics, Paris, France, Paris, France; 4 Institut Pasteur, Université Paris Cité, Biodiversity and Epidemiology of Bacterial Pathogens, F-75015, Paris, France; 5 National Hospital of Tropical Diseases, Hanoi, Vietnam; 6 Department of Biochemistry & Molecular Biology, Centre for Systems Genomics, University of Melbourne, Melbourne, Australia; 7 Centre for Tropical Medicine and Global Health, Nuffield Department of Medicine, University of Oxford, Oxford, UK; 8 Department of Medical Microbiology and Radboudumc Center for Infectious Diseases, Radboudumc, Nijmegen, The Netherlands

**Keywords:** Klebsiella pneumoniae, K64, capsule type, community-acquired infections, carbapenem-resistant

## Abstract

**Introduction:**

Recent reports indicate the emergence of community-acquired pneumonia associated with K64- *Klebsiella pneumoniae (K. pneumoniae)*. Here, we identify the capsular types and sequence type of invasive and commensal *K. pneumoniae* isolates from Vietnam.

**Methods:**

We included 93 *K. pneumoniae* isolates from patients hospitalized at the National Hospital for Tropical Diseases, Hanoi between 2007 and 2011; and 110 commensal isolates from throat swabs from healthy volunteers living in rural and urban Hanoi in 2012. We determined sequence types (STs) by multi-locus sequence typing (MLST) and capsule typing for seven K types by PCR. Antibiotic susceptibility testing was performed using disk diffusion.

**Results:**

The most common detected capsule types were K1 (39/203, 19.2%, mainly ST23) and K2 (31/203, 15.3%, multiple STs: ST65, ST86, ST380). We found significantly more K2 isolates among invasive in comparison to commensal isolates (22.6% vs 9%, p = 0.01) but no significant difference was observed between invasive and commensal K1 isolates (14.5% vs 24.7%, p = 0.075). K64 with varying sequence types were predominantly seen among invasive *K. pneumoniae *(8 vs. 3) and were isolated from sepsis and meningitis patients. Among K64 isolates, one was carbapenem-resistant with ST799.

**Conclusion:**

Our study confirms that capsule type K64 *K. pneumoniae* is associated with community-acquired invasive infections in Vietnam. Research is needed to unravel the mechanisms of virulence of capsule type K64 in both community and hospital settings.

## Introduction

In low and middle-income countries in Asia, like Vietnam, *K. pneumoniae* is an important cause of severe community-acquired infections, including pneumonia, liver abscesses and sepsis. ^1^ Multidrug-resistance in *K. pneumoniae*, especially among hospital acquired infections, is an emerging problem associated with high morbidity and mortality. ^2^ *K. pneumoniae* is classified across two main virulence phenotypes, classical (cKp) and hypervirulent (hvKp). Most *K. pneumoniae* are associated with uncomplicated community acquired infections and nocosomial infections. However, hvKp results in more severe community – acquired infections with manifestations such as pyogenic liver abscess, meningitis, endophthalmitis, and necrotizing fasciitis. ^3^ The polysaccharide capsule is perhaps the most well-known virulence factor of K. pneumoniae, including for the hvKp phenotype. The capsule surrounding *K. pneumoniae* cells can be divided into at least 79 capsular types (K1 to K79), among which K1, K2, and K64 are common serotypes of the MDR-hvKp group, which is commonly found in Asia. ^4^^,^^5^ A genomic analysis of diversity and population structure of 288 human and animal *K. pneumoniae* isolates from six countries, spanning four continents, has shown that K64 was among the important capsule types associated with community acquired pneumonia in Vietnam (n = 3) and Singapore (n = 1). ^5^ In addition to cases and outbreaks reported on severe *K. pneumoniae* infections by K64 capsular type with the convergence of carbapenem-resistant phenotypes, ^6^ in one case report, K64-ST1764 *K. pneumoniae* was found to be a cause of pyogenic liver abscess and endogenous endophthalmitis. *K. pneumoniae* can asymptomatically colonize the gastrointestinal (proportion between 40% to 66%) ^7^ and upper respiratory tract of healthy humans (14.1%) ^8^ but carriage of K64 *K. pneumoniae* in healthy persons has rarely been described. According to previous studies, K1 and K2 are known to be highly virulent capsule types associated with community-acquired and hospital-acquired infections. K54 and K57 are mainly found in the human microbiome, particularly in the upper respiratory tract of healthy individuals. Our literature review indicates that K5 and K20 have been sporadically reported as pathogens, although they are less common. As for K64, reported by K. Holt et al. in 2015, it appears to be an emerging cause of severe disease. ^5^^,^^9^ Our study aims to investigate the diversity of *K. pneumoniae* in both community and hospital settings by classifying the diversity of capsular types. Here, we found K64- *K. pneumoniae* to be more common among invasive isolates as compared to commensal isolates isolated from Vietnamese individuals.

## Methods

### *Klebsiella pneumoniae* isolates and antibiotic susceptibility testing

From a total of 589 *K. pneumoniae* that were isolated from patients hospitalized at the National Hospital of Tropical Diseases between 2007 and 2011, 332 isolates were recovered after re-cultivation. Of those, 30 isolates with lacking clinical metadata were excluded, leaving the remaining 302 isolates for downstream analysis. Ninety-three were isolated from otherwise sterile sites, including blood (n = 70), cerebrospinal fluid (CSF) (n = 7), and pus (n = 16). These were re-cultured and re-confirmed using biochemical test strips (API 20E, Biomérieux, Marcy l’Étoile, France). Antibiotic susceptibility testing (AST) using disk diffusion was done according to Clinical and Laboratory Standards Institute (CLSI) guidelines 2019. A phenotypic confirmatory double-disk test was performed for confirmation of ESBL production using CTX (30 mg) and CAZ (30 mg) disks alone and in combination with CA (10 mg) (Mast Diagnostic a GmbH, Reinfeld, Germany).

We collected clinical data from patients infected with these isolates (invasive isolates) and classified their infections based on the definition of healthcare-associated and community-associated infections ( https://arpsp.cdc.gov/profile/infections?tab=nhsn). To ensure that the isolates were from community-acquired infections, we used a time difference of ≥48 hours between the time of admission and the time of specimen collection. To compare invasive with commensal isolates, we used randomization tools ( https://www.randomizer.org/) to select 110 of 331 *K. pneumoniae* isolates from throat swabs of healthy volunteers living in rural (Bavi) and urban (DongDa district), Hanoi in 2012. The epidemiology of these healthy volunteers has been described in our previous study which was designed to investigate *K. pneumoniae* oropharyngeal carriage and rick factors in Vietnam. ^8^ Commensal isolates were tested and analysed in the same manner as invasive isolates.

### Molecular typing

Invasive and commensal isolates were tested to identify their capsule types (for capsule types K1, K2, K5, K20, K54, K57, and K64) by polymerase chain reaction (PCR) according to previously described methods. ^10, 11^ A specific K64 PCR was developed to detect capsule type K64 that was reported to be common in Southeast Asia ^5^ with the following primers: Forward (5′TTC TTT AAG TCT TCT GGG TAT CA3′) and Reverse (5′AGT CTT TAA TCG CCT TCT3′). The PCR cycling program for K64 consisted of 95°C for 15 min, followed by 30 cycles of 95 °C for 30 sec, 60 °C for 30 sec, 72 °C for 1 min 20 sec and the final elongation step was performed for 7 min at 72 °C. The PCR products were loaded on agarose (1.5%) gel electrophoresis. Samples contained PCR products with size equivalent to 782 bp as K64 positive.

Multi-locus sequence typing (MLST) was performed by sequencing the PCR products of seven house-keeping genes including (gapA, infB, mdh, pgi, phoE, rpoB, tonB). The sequence of these genes was analysed using the BIGSdb-Pasteur website ( https://bigsdb.pasteur.fr/) for determining the sequence types. Sequence types (STs) were grouped into clonal complexes (CC) as described previously. ^12^ A clonal complex is defined as a group of STs with at least 6 identical alleles with at least one other member of the group. STs that did not fall within a CC were defined as singletons.

We used Statistical Package of Social Sciences (SPSS) version 25 (IBM corporation, Armonk (NY), USA) for analysis, ^13^ p values < 0.05 were considered significant (2-sided).

### Ethics statement

This study was approved by the Oxford University Tropical Research Ethics Committee (Oxtrec, 49-12) and the National Hospital for Tropical Diseases Institutional Review Board. Before participation, written informed consent from subjects or, in case of minors, their caregivers, was obtained on a standard study consent form.

## Results

Among 203 *K. pneumoniae* isolates, 100 (49.2%) were positive with one of the seven tested capsule (K) types (K1, K2, K5, K20, K54, K57, K64). The most common K types were K1 (n = 39) and K2 (n = 31). Whereas 36/39 (92.3%) K1 isolates belonged to STs that were classified into clonal complex, CC23, K2 isolates were more diverse: the most frequent clonal complex was CC65 (n = 18), followed by CC86 (n = 8) ( Table 1). While K2 isolates were more prevalent among invasive than among commensal isolates (22.6% vs 9%, Chi-square, p = 0.01), K1 was relatively equally distributed (14.5% vs 24.7%, p = 0.075), and K57 (n = 18) was detected mostly among commensal isolates (15.4% vs 1%, p < 0.0001). We detected seven isolates with K64, five of which were invasive (p < 0.001). Among five invasive K64 *K. pneumoniae*, two were isolated from sepsis patients, one from meningitis, one from sepsis-meningitis, and one from the blood of a patient with hospital-acquired pneumonia. Most of these invasive K64 isolates (4/5) were from patients on Intensive Care Units (ICU). Of those patients, two had fatal community acquired pneumonia. The seven K64 isolates (two from commensal, five from invasive isolates) were genotyped by MLST: four belonged to the CC231 (ST231, ST799, ST807) and the other to CC65 (ST692).

**Table 1.** Clonal complex (CCs) as determined by multi-locus sequence typing (MLST) and distribution of capsular types among invasive isolates and commensal isolates of *Klebsiella pneumoniae* in Vietnam. P-value were determined by Chi-square (2-sides) test.

Clonal Complex (CC)	Overall	Commensal (n, %)	Invasive (n, %)	p-value
23	63 (31)	35 (31.8)	28 (28)	0.791
65	23 (11.3)	6 (5.5)	17 (18.2)	0.004
231	4 (2)	0 (0)	4 (4.3)	
412	9 (4.4)	8 (7.3)	1 (1)	
806	6 (3)	1 (1)	5 (5.3)	
86	8 (4)	3 (2.7)	5 (5.3)	
Others CCs	23 (11.3)	15 (13.6)	7 (7.5)	0.251
Singleton	68 (34)	42 (38.1)	26 (27.9)	0.083
**Capsular type**				
K1	39 (19.2)	16 (14.5)	23 (24.7)	0.075
K2	31 (15.3)	10 (9)	21 (22.6)	0.01
K5	1 (0.5)	0 (0)	1 (1)	0.458
K20	2 (1)	2 (1.8)	0 (0)	0.5
K54	2 (1)	1 (1)	1 (1)	1
K57	18 (9)	17 (15.4)	1 (1)	<0.0001
K64	7 (3.4)	2 (1.8)	5 (5.4)	<0.0001

Overall, antimicrobial resistant proportions of commensal isolates differed significantly from invasive *K. pneumoniae* ( Table 2). Among K64 isolates, one invasive ST799 isolate from a patient with hospital-acquired pneumonia was multi-drug resistant, with resistance to imipenem, ciprofloxacin, trimethoprim/sulfamethoxazole, piperacillin-tazobactam and gentamicin. Of the remaining K64 isolates, four invasive isolates were non-carbapenem resistant but they either were resistant to trimethoprim/sulfamethoxazole or piperacillin-tazobactam. Whilst, the two commensal isolates with ST1331 and ST1347, were susceptible to all tested antibiotics. ^14^

**Table 2.** Comparison of the proportion of antibiotic resistance between invasive versus commensal *Klebsiella pneumoniae *isolates in Vietnam. P-value were determined by Chi-square (2-sides) test.

	Overall	Commensal (n, %)	Invasive (n, %)	p-value
ESBL	15 (7.4)	4 (3.6)	11 (11.8)	0.025
CIP (Ciprofloxacin)	7 (3.4)	0 (0)	7 (7.5)	0.002
AMC (Amoxicillin - clavulanate)	16 (7.8)	0 (0)	16 (17.2)	0.004
AMP (Ampicillin)	196 (96.5)	104 (94.5)	92 (98.5)	0.339
FEP (Cefepime)	9 (4.4)	0 (0)	9 (9.6)	0.001
GEN (Gentamicin)	N/A	N/A	14 (15)	N/A
TZP (Piperacillin -tazobactam)	6 (2.9)	0 (0)	6 (6.5)	0.008
SXT (Trimethoprim-sulfamethoxazole)	29 (14.3)	9 (8.1)	20 (21.5)	<0.001
IMP (Imipenem)	1 (0.5)	0 (0)	1 (1)	0.458

## Discussion and conclusion

In addition to the emergence of carbapenem-resistant *K. pneumoniae* worldwide, previous studies have shown that infections caused by hypervirulent carbapenem susceptible *K. pneumoniae* can also be considered a threat to public health. ^15^ Our study suggested that besides capsule type K2, capsule type K64 was associated with invasive strains (5.4% vs 1.8%, Chi-square, p < 0.001), consistent with previous studies. ^5, 16^

The capsular type K64 has been reported worldwide, especially from clinical settings in Asia, Europe, and North America and is one of the K types associated with hypervirulent strains, that have shown simultaneous expression of virulence and carbapenem-resistance genes, posing a treatment challenge. ^17^ ST11-K64 is a common type in China, possibly leading to pyogenic liver abscesses. ^9^ Contrarily, our K64 strains were mainly found in sepsis and meningitis patients with varying STs, including: ST231, ST692, ST799, and ST807. Moreover, it is worth noting that K64 has been common in *Klebsiella pneumoniae* carbapenemase (KPC) producing ST11 strains in China, and the shift from K47 to K64 has been associated with increased virulence in this strain. ^18^ In our study, the carbapenem-resistant K64-ST799 was isolated from the blood of a hospital-acquired patient in 2011, and was not detected in subsequent years in surveillance efforts. ^19^ Likely, the K64-ST799 strain might have acquired a mobile element carrying a carbapenemase-producing gene.

In particular, K64 has been recently recognized as a capsular type potentially associated with hypervirulence and invasive disease. Indeed, the presence of K64 with several STs isolated from bacteraemia and meningitis patients in Vietnam and a pyogenic liver abscess patient in China ^20^ provides further evidence that strains with this capsule type are virulent. ^21^

The present study has several limitations. Because of the retrospective nature of the analyzed data collection, we missed some clinical data (exposures, alcohol history, out come after treatment) of the patients. The results of this study lack evidence to support the hypothesis that the risk factor of infections may be *K. pneumoniae* colonizers. In this study, the relatively small number of K64 isolates provided only limited data about the serotype's convergent virulence and carbapenem resistance. Also, we lack the whole genome sequence data of these K64 isolates for further understanding the molecular basis of hypervirulence and the phylogenetic positioning of this rare ST within the genomic taxonomy of *K. pneumoniae*. ^22^ However, our results led support to the hypothesis that K64 is associated with severe invasive community acquired *K. pneumoniae* infections, including sepsis and meningitis. In addition, here, only seven capsule types were selected to investigate the diversity of *K. pneumoniae* isolates. We recognize that this is one of the limitations of our study. Further studies are needed to unravel the mechanisms of virulence of capsule type K64 in *K. pneumoniae.*

## Data availability

### Underlying data

Dryad: *Klebsiella pneumoniae* with capsule type K64 is overrepresented among invasive disease in Vietnam. https://doi.org/10.5061/dryad.h44j0zpjv ^14^

• Table 1 (Kp_All_AST) provides the detailed information of 203 *Klebsiella pneumoniae* isolates including source of isolates, date of collection, antibiotic susceptibility profiles, K-serotypes and MLST profiles.
• Serotype_Clinical isolates.rar and Serotype_Community isolates.rar: These folders contain the photographs of the PCR products of agarose gel electrophoresis. Maps of samples on agarose plates are described in two Excel files (Electrophoresis_map.xlsx and Isolate ID on Electrophoresis.gel.xlsx for Clinical isolates and Commensal isolates, respectively).

Data are available under the terms of the Creative Commons Zero “No rights reserved” data waiver (CC0 1.0 Public domain dedication).

## Consent

Before participation, written informed consent from subjects or, in case of minors, their caregivers, was obtained on a standard study consent form.
