# Supplementary material for: Klebsiella pneumoniae with capsule type K64 is overrepresented among invasive disease in Vietnam
Source: F1000Res. 2025 Jan 17;10:454. Originally published 2021 Jun 8. [Version 2] doi: 10.12688/f1000research.52799.2 (PMC12335726; doi:10.12688/f1000research.52799.2)
Supplement: Peer review history

# Reviewer report: Reviewer response for version 2

**DOI:** 10.5256/f1000research.176630.r393103

**Published:** 2025-08-09

**Authors:** Neris García-González

**Affiliations:** 1 Instituto de Biomedicina de Valencia (Ringgold ID: 54426), Valencia, Valencian Community, Spain

Peer Review for “ *Klebsiella pneumoniae* with capsule type K64 is overrepresented among invasive disease in Vietnam [version 2]”

 The manuscript explores the distribution of *Klebsiella pneumoniae* capsule types, with a focus on K64, among invasive and commensal isolates in Vietnam. While the topic is relevant and the data valuable, I believe the conclusions are overstated given the limited number of K64 isolates. 

 Several aspects of the methodology and analysis require clarification or revision, particularly to address the overstatement of key conclusions. Additionally, the overall writing—especially the methods section—would benefit from careful editing for clarity.

**Major Comments**

1.  The conclusion that “Our study confirms that capsule type K64 *K. pneumoniae* is associated with community-acquired invasive infections in Vietnam” is, in my view, overstated given the data presented. Of the 100 isolates with a successfully determined capsular type, only 11 were K64 (5 from invasive, 2 from commensal samples). This is a very small number, and the application of statistical tests to such low counts does not support a definitive conclusion. 

Similarly, the claim that K64 is “associated with sepsis and meningitis” is unsupported. While some K64 isolates came from such cases, no analysis was done to test specific disease associations. This should be rephrased more cautiously (e.g., “were observed in”).

1. The statement that “K64-ST799 might have acquired a mobile element carrying a carbapenemase gene” is speculative and not backed by molecular data. Without sequencing or resistance gene analysis, this claim should be softened.
2. The authors tested for only seven capsule types (K1, K2, K5, K20, K54, K57, K64). It’s unclear why these were chosen—are they the most common in Vietnam, or just associated with hvKp globally? This should be clarified, especially since over half of the isolates were untypeable. Without knowing whether additional relevant types were missed, it is difficult to interpret the significance of the K64 frequency.
3. The invasive isolates were collected between 2007–2011, while the commensal isolates were collected in 2012. The invasive and commensal isolates were collected in different time periods (2007–2011 vs. 2012), which could introduce temporal bias. This limitation should be acknowledged explicitly.

The manuscript requires substantial revision for clarity. Several sentences are difficult to parse, and the structure of some sections hinders comprehension. For example:

- The methodology does not clearly explain how the 589 *K. pneumoniae* isolates were selected. Were these all from invasive infections? If so, and only 49.2% were typeable, a large proportion of relevant invasive capsule types may have been missed. Alternatively, if this set included both invasive and commensal isolates, it would improve the comparison by providing community data from the same time period. This should be clearly stated and clarified in the manuscript.
- The distinction between the two datasets (invasive and commensal) is clear in the abstract but not well explained in the main text. I suggest rephrasing to something similar to: “To compare invasive with commensal isolates, we included commensal isolates from a previous study. These were throat swabs from healthy volunteers in rural and urban Hanoi in 2012. …”
- The statement: “To ensure that the isolates were from community-acquired infections, we used a time difference of ≥48 hours…” is confusing. Community-acquired infections are typically defined as those occurring within 48 hours of hospital admission. This should be reworded for clarity.

**Minor Comments**

- Define MDR (multidrug-resistant) on first mention.
- In Tables 1 and 2, “Overall” column should also have (n, %)
- Correct spelling of “nocosomial” to “nosocomial.”

Is the work clearly and accurately presented and does it cite the current literature?

Partly

If applicable, is the statistical analysis and its interpretation appropriate?

Partly

Are all the source data underlying the results available to ensure full reproducibility?

No source data required

Is the study design appropriate and is the work technically sound?

Partly

Are the conclusions drawn adequately supported by the results?

Partly

Are sufficient details of methods and analysis provided to allow replication by others?

Yes

Reviewer Expertise:

microbial genomics

I confirm that I have read this submission and believe that I have an appropriate level of expertise to confirm that it is of an acceptable scientific standard, however I have significant reservations, as outlined above.

# Reviewer report: Reviewer response for version 2

**DOI:** 10.5256/f1000research.176630.r383866

**Published:** 2025-06-06

**Authors:** Ning Dong

**Affiliations:** 1 Zhejiang University School of Medicine, Hangzhou, China

In this study on *Klebsiella pneumoniae* , the authors analyzed 203 clinical isolates and found that capsular K64 was significantly enriched in invasive infections, mainly associated with sepsis and meningitis, and some K64 strains (such as ST799) were multidrug resistant. This study suggests that K64 is an important pathogen of community-acquired invasive infections in Vietnam. I suggest the following directions for further improvement of the study:

 The theoretical basis for capsule type selection was supplemented, and the latest literature (such as studies on the global prevalence of K64 after 2023) was cited to complete the discussion section.

 The sample size was small and underrepresented. The sample size of K64 strains was only 7 strains (5 invasive strains and 2 commensal strains), which was too small to support the conclusion that K64 was significantly enriched in invasive infections, which may lead to limited reliability of statistical results. Second, the commensal strains lack sample coverage from multiple regions and time points to fully reflect the epidemiological characteristics of community-acquired infections in Vietnam. 

 The results "K64 with varying sequence types were predominantly seen among invasive *K. pneumoniae* (8 vs. 3)" was inconsistent with the table data in the text (5 invasive strains, 2 commensal strains).

 The whole genome sequencing data was supplemented to analyze the virulence genes (such as *rmpA* and *magA* ) and drug resistance genes (such as *KPC* and *NDM* ) of K64 strain, and to clarify its pathogenic mechanism at the molecular level.

 There is a logical jump between the conclusion and the data. The title and conclusion use the word "overrepresented", which is misleading. Because the proportion of K64 in invasive strains was only 5.4% (5/93), which was much lower than that of K1 (24.7%) and K2 (22.6%). Second, it was mentioned that K64 was associated with carbapenem resistance (such as the ST799 strain). However, the finding of only one drug-resistant strain cannot support the inference that the K64 strain is generally resistant to carbapenem, and more data are needed to verify it.

Is the work clearly and accurately presented and does it cite the current literature?

Partly

If applicable, is the statistical analysis and its interpretation appropriate?

Yes

Are all the source data underlying the results available to ensure full reproducibility?

Yes

Is the study design appropriate and is the work technically sound?

Yes

Are the conclusions drawn adequately supported by the results?

Partly

Are sufficient details of methods and analysis provided to allow replication by others?

Yes

Reviewer Expertise:

Epidemiology of infectious diseases

I confirm that I have read this submission and believe that I have an appropriate level of expertise to state that I do not consider it to be of an acceptable scientific standard, for reasons outlined above.

# Reviewer report: Reviewer response for version 2

**DOI:** 10.5256/f1000research.176630.r360165

**Published:** 2025-01-28

**Authors:** Frank R. DeLeo

**Affiliations:** 1 Laboratory of Bacteriology, Rocky Mountain Laboratories, National Institute of Allergy and Infectious Diseases, National Institutes of Health, Hamilton, MT, 59840, USA

Revised version is appropriate.

Is the work clearly and accurately presented and does it cite the current literature?

Partly

If applicable, is the statistical analysis and its interpretation appropriate?

I cannot comment. A qualified statistician is required.

Are all the source data underlying the results available to ensure full reproducibility?

Partly

Is the study design appropriate and is the work technically sound?

Yes

Are the conclusions drawn adequately supported by the results?

Partly

Are sufficient details of methods and analysis provided to allow replication by others?

Partly

Reviewer Expertise:

Bacterial pathogenesis and host defense. Primary focus on Staphylococcus aureus, Klebsiella pneumoniae, and human neutrophil biology.

I confirm that I have read this submission and believe that I have an appropriate level of expertise to confirm that it is of an acceptable scientific standard.

# Reviewer report: Reviewer response for version 1

**DOI:** 10.5256/f1000research.56121.r287539

**Published:** 2024-06-28

**Authors:** Frank R. DeLeo

**Affiliations:** 1 Laboratory of Bacteriology, Rocky Mountain Laboratories, National Institute of Allergy and Infectious Diseases, National Institutes of Health, Hamilton, MT, 59840, USA

Ngoc et al. report capsule type and multilocus sequence type of *Klebsiella pneumoniae *clinical isolates from patients hospitalized at National Hospital for Tropical Diseases in Hanoi from 2007 to 2011. The authors compared these isolates to commensal isolates obtained by throat swab from healthy volunteers in 2012. The study is interesting and adds to our understanding of the distribution of the *K. pneumoniae* capsule types associated with human infections. I have a few comments for the authors to consider.

1. Please verify numbers of isolates in Table 1 compared with what is stated in the text. By my count, there are 110 commensal isolates (correct in the text) and 94 (stated as 93 in the text) invasive isolates. These numbers are concordant with the sum of the numbers in the Overall column, which total 204 isolates (the text indicates 203). Please verify for accuracy.
2. It would be optimal to provide text in the Table 1 legend that states what the p-value refers to from a comparison standpoint, and indicate the test used here as well.
3. I recommend providing the rationale for selecting the seven capsule types used to screen isolates in this study. Why were these seven capsule types chosen among others?
4. A conclusion of the study is that “capsule type K64 is overrepresented among invasive strains”. In reality there are only seven K64 isolates out of 204 total isolates and only five of these were from invasive disease. This is 5.4% of the total number of invasive isolates and much less than either K1 or K2. In addition, the *Conclusion *text on the title page states that K64 *K. pneumoniae* is associated with community-acquired invasive infections in Vietnam.” Please provide the criteria in the Methods section that were used to determine whether an infection is a bona fide community-acquired infection. It would be fair to state that K64 contributes to invasive disease and is among the top three capsule types tested that are associated with invasive infections. However, the term “overrepresented among invasive strains” might best be restated as “there were significantly more capsule type K64 isolates recovered from patients with invasive infections compared to healthy individuals with commensal isolates” or something along those lines.

Minor points.   

 Introduction: “A genomic analysis of diversity and population structure of 288 human and animal *K. pneumoniae* isolates from six countries, spanning four continents, has shown that K64 mostly found in Vietnam (n = 3) and Singapore (n = 1), was among the important capsule types associated with community acquired pneumonia.” is not easy to read.

 Consider revising to:

 “A genomic analysis of diversity and population structure of 288 human and animal *K. pneumoniae* isolates from six countries, spanning four continents, has shown that K64 was among the important capsule types associated with community acquired pneumonia in Vietnam (n = 3) and Singapore (n = 1).”

 Introduction, penultimate sentence: change K64 capsular to K64 capsule types

Is the work clearly and accurately presented and does it cite the current literature?

Partly

If applicable, is the statistical analysis and its interpretation appropriate?

I cannot comment. A qualified statistician is required.

Are all the source data underlying the results available to ensure full reproducibility?

Partly

Is the study design appropriate and is the work technically sound?

Yes

Are the conclusions drawn adequately supported by the results?

Partly

Are sufficient details of methods and analysis provided to allow replication by others?

Partly

Reviewer Expertise:

Bacterial pathogenesis and host defense. Primary focus on Staphylococcus aureus, Klebsiella pneumoniae, and human neutrophil biology.

I confirm that I have read this submission and believe that I have an appropriate level of expertise to confirm that it is of an acceptable scientific standard, however I have significant reservations, as outlined above.

# Reviewer report: Reviewer response for version 1

**DOI:** 10.5256/f1000research.56121.r93803

**Published:** 2021-10-12

**Authors:** John L. Kiley

**Affiliations:** 1 Brooke Army Medical Center, San Antonio, TX, USA

This paper describes the epidemiology of K64 *Klebsiella pneumoniae *isolates with a particular focus on K64 from a hospital in Vietnam.

**Introduction**

- "...but K64 capsular have rarely been described in healthy carriers." in the introductory paragraph reads a bit unclearly.
- Adding a bit more about the importance of K64 and the differences between classic and hypervirulent types of *Klebsiella *spp. (as well as how capsules might play a role in this) would better situate the authors' opening argument as to why they are performing this current study.  

**Methods**

- "302 *K. pneumoniae *were isolated..." are these all isolates from the hospital during this time period?

**Results**

- I note that ESBL production was determined in Table 2 - please clarify how you determined this in the methods.

**Discussion and conclusion**

- I think some discussion about the small numbers of K64 isolates would also be helpful in the limitations of the paper.  The second paragraph starts off by suggesting K64 has "been little reported so far," but I think the data from China would argue against this.
   
  - Zhang *et al*., 2020 reported Chinese epidemiological data on K64 ^1^.
  - Walker *et al*., 2020 for general discussion of hypervirulence and association with capsular type ^2^.
  - Catalán-Nájera *et al*., 2017 for hypermucoviscious, hypervirulent differences/discussion ^3^.

Great paper overall!

Is the work clearly and accurately presented and does it cite the current literature?

Partly

If applicable, is the statistical analysis and its interpretation appropriate?

Yes

Are all the source data underlying the results available to ensure full reproducibility?

Yes

Is the study design appropriate and is the work technically sound?

Yes

Are the conclusions drawn adequately supported by the results?

Yes

Are sufficient details of methods and analysis provided to allow replication by others?

Yes

Reviewer Expertise:

Multi drug resistant Gram-negative infections

I confirm that I have read this submission and believe that I have an appropriate level of expertise to confirm that it is of an acceptable scientific standard, however I have significant reservations, as outlined above.

## References

[1] : Evolution of hypervirulence in carbapenem-resistant Klebsiella pneumoniae in China: a multicentre, molecular epidemiological analysis. *Journal of Antimicrobial Chemotherapy* .2020;75(2) : 10.1093/jac/dkz446 327-336 10.1093/jac/dkz446 31713615

[2] : The intersection of capsule gene expression, hypermucoviscosity and hypervirulence in Klebsiella pneumoniae. *Curr Opin Microbiol* .54: 10.1016/j.mib.2020.01.006 95-102 10.1016/j.mib.2020.01.006 32062153 PMC8121214

[3] : Hypervirulence and hypermucoviscosity: Two different but complementaryKlebsiella spp. phenotypes?. *Virulence* .2017;8(7) : 10.1080/21505594.2017.1317412 1111-1123 10.1080/21505594.2017.1317412 PMC571139128402698

# Response

**Published:** 2025-01-04

**Authors:** Bich Vu Thi Ngoc

**Affiliations:** Oxford University Clinical Research Unit - Hanoi, Vietnam

We would like to thank reviewer for your valuable comments. We have addressed all the reviewers’ comments in the revised manuscript, including with additional information, and responded in the point-to-point rebuttal below. We hope our revisions are adequate and make our manuscript clear and publishable.

**Reviewer 1**

 This paper describes the epidemiology of K64 *Klebsiella pneumoniae* isolates with a particular focus on K64 from a hospital in Vietnam.

**Comment 1**: Introduction

 "...but K64 capsular have rarely been described in healthy carriers." in the introductory paragraph reads a bit unclearly.

**Response**:  Thank you very much for your comment. We have now revised the sentence in the updated version as below:

 “…but carriage of K64 *K. pneumoniae *in healthy persons has rarely been described.”

**Comment 2:**

 Adding a bit more about the importance of K64 and the differences between classic and hypervirulent types of Klebsiella spp. (as well as how capsules might play a role in this) would better situate the authors' opening argument as to why they are performing this current study. 

**Response:** Thank you very much for your recommendation. We have now included the following sentences in the introduction of the manuscript:

*K pneumoniae* is classified across two main virulence phenotypes, classical (cKp) and hypervirulent (hvKp). Most *K. pneumoniae* are associated with uncomplicated community acquired infections and nocosomial infections. However, hvKp results in more severe community – acquired infections with manifestations such as pyogenic liver abscess, meningitis, endophthalmitis, and necrotizing fasciitis. The polysaccharide capsule is perhaps the most well-known virulence factor of *K. pneumoniae*, including for the hvKp phenotype. The capsule surrounding *K. pneumoniae* cells can be divided into at least 79 capsular types (K1 to K79), among which K1, K2, and K64 are common serotypes of the MDR-hvKp group, which is commonly found in Asia.

**Comment 3:** Methods

 "302 *K. pneumoniae* were isolated..." are these all isolates from the hospital during this time period?

**Response:**

 Thank you very much for your question. The total number of isolates from the hospital during this time was 589 isolates; however, because this was a retrospective study, we performed re-culture, and 332/589 isolates were recovered. Of those, 30 isolates had no associated clinical metadata, and were excluded from the study.

 We now have clarified this in the manuscript as below:

 “From a total of 589 *K. pneumoniae* that were isolated from patients hospitalized at the National Hospital of Tropical Diseases between 2007 and 2011, 332 isolates were recovered after re-cultivation. Of those, 30 isolates with lacking clinical metadata were excluded, leaving the remaining 302 isolates for downstream analysis.”

**Comment 4:** Results

 I note that ESBL production was determined in Table 2 - please clarify how you determined this in the methods.

**Response:**

 Thank you very much for your comment. We have added the information into the updated version as below:

 “A phenotypic confirmatory double-disk test was performed for confirmation of ESBL production using CTX (30 mg) and CAZ (30 mg) disks alone and in combination with CA (10 mg) (Mast Diagnostic a GmbH, Reinfeld, Germany).”

**Comment 5:** Discussion and conclusion

 I think some discussion about the small numbers of K64 isolates would also be helpful in the limitations of the paper.  The second paragraph starts off by suggesting K64 has "been little reported so far," but I think the data from China would argue against this.

**Response:** Thank you for your recommendation.

 We agree that data from China would argue against our statement in the second paragraph starts off by suggesting K64 has "been little reported so far,". We now have revised the sentence as below:

 “The capsular type K64 has been reported worldwide, especially from clinical settings in Asia, Europe, and North America and is one of the K types associated with hypervirulent strains, that have shown simultaneous expression of virulence and carbapenem-resistance genes, posing a treatment challenge.”

 Regarding the small numbers of K64 isolates in our study, we have added into the limitation:

 “In this study, the relatively small number of K64 isolates provided only limited data about the serotype's convergent virulence and carbapenem resistance. Also, we lack the whole genome sequence data of these K64 isolates for further understanding of the molecular basis of hypervirulence and the phylogenetic positioning of this rare ST within the genomic taxonomy of *K. pneumoniae*. However, our results led support to the hypothesis that K64 is associated with severe invasive community acquired *K. pneumoniae* infections, including sepsis and meningitis. In addition, here, only seven capsule types were selected to investigate the diversity of *K. pneumoniae* isolates. We recognize that this is one of the limitations of our study. Further studies are needed to unravel the mechanisms of virulence of capsule type K64 in *K. pneumoniae*.”
